# Photocatalytic Solar Tower Reactor for the Elimination of a Low Concentration of VOCs

**DOI:** 10.3390/molecules191016624

**Published:** 2014-10-15

**Authors:** Nobuaki Negishi, Taizo Sano

**Affiliations:** Research Institute for Environmental Management Technology, National Institute of Advanced Industrial Science and Technology, 16-1 Onogawa, Tsukuba 305-8569, Japan

**Keywords:** photocatalyst, VOCs, toluene, solar reactor, field test

## Abstract

We developed a photocatalytic solar tower reactor for the elimination of low concentrations of volatile organic compounds (VOCs) typically emitted from small industrial establishments. The photocatalytic system can be installed in a narrow space, as the reactor is cylindrical-shaped. The photocatalytic reactor was placed vertically in the center of a cylindrical scattering mirror, and this vertical reactor was irradiated with scattered sunlight generated by the scattering mirror. About 5 ppm toluene vapor, used as representative VOC, was continuously photodegraded and converted to CO_2_ almost stoichiometrically under sunny conditions. Toluene removal depended only on the intensity of sunlight. The performance of the solar tower reactor did not decrease with half a year of operation, and the average toluene removal was 36% within this period.

## 1. Introduction

Photochemical oxidants, mainly volatile organic compounds (VOCs), are currently a serious global environmental problem, particularly in large cities. In Japan, approximately 10% of VOCs are emitted from automobiles, while the rest are of fixed origin [1,2]. Another major source of VOC emissions (approximately 73%) is solvent from paint and printing industries [2]. Installation of a VOC elimination system is certainly possible at some fixed emission sources, and a few industry groups are undertaking its implementation. On the other hand, smaller industrial establishments with VOC emissions below the average level in Japan are under no such obligations, although self-imposed regulation is recognized. However, most establishments cannot install a VOC elimination system, mainly for economic reasons. Small industrial establishments are the main contributors to VOC emissions in Japan; for instance, these are the source of around 70% of VOC emissions in Tokyo [3]. Thus, minimization of VOC emissions from these facilities would be the most effective approach to eliminate this photochemical oxidant.

Practical application of photocatalysis by TiO_2_ to self-cleaning materials has begun [4]; on the other hand, application to air purification is still not widely familiar, with the exception of a few examples, such as photocatalytic pavement blocks for the removal of nitrogen oxides (NOx) [5,6]. However, the application of TiO_2_ photocatalysts for VOC elimination is also of interest. This is a simple process compared to NOx removal, since the final products are generally CO_2_ and H_2_O, which do not poison the surface of the photocatalyst. In addition, a few methods for the elimination of VOCs by photocatalytic materials have already been standardized (ISO 22197-2, ISO 22197-3) [7,8].

Photocatalysis can be activated by solar irradiation, which would enable continuous elimination of VOCs. Therefore, the combination of photocatalysis and solar energy would reduce the electric energy required for the elimination of VOCs and, consequently, the operational costs. While this method would be unsuitable for high concentrations of VOCs and/or high airflow, its application to small industrial establishments would be advantageous. That is, the amount of VOC emissions from these establishments is rather small; thus, the installation of a photocatalytic system would be suitable for such an emission source with a low VOC level and flow rate.

Various types of photocatalytic solar reactors have been recently proposed. Standard types of solar reactors are mainly parabolic trough or flat panel, and a few researchers have already investigated the optimum design to achieve maximum efficiency [9,10,11,12,13]. However, the design of solar reactors is limited to water treatment thus far, and that for air treatment has not received as much attention [14]. The discussion above explained the state of VOC emissions in Japan. In the case of a megalopolis, such as Tokyo or Osaka, small industrial establishments are cramped within a narrow space; therefore, a flat- or trough-type photoreactor would be difficult to set up for these establishments. In addition, expensive instruments, such as a solar tracking system for a solar photoreactor, are difficult to install [15]. Therefore, we developed a unique photocatalytic system consisting of a tower-like photoreactor with a lens barrel that would be suitable for installation in a narrow space, such as the gap between buildings. The performance of this photocatalytic solar tower reactor per unit area of photocatalyst is possibly lower than that of a flat- or trough-type photoreactor, because the surface exposed to solar radiation is limited to the cross-sectional area of the tower. However, the structure of this reactor is very simple, and this will reduce the costs of production, installation, operation and maintenance. We expect that it will be possible in the near future to eliminate photochemical oxidant generation under sunny conditions and to decrease VOC emission by extensively installing solar tower reactors in the areas populated by small industrial establishments. In this study, we describe the design and construction of the solar tower reactor and evaluate the VOC elimination performance through long-term experiments.

## 2. Results and Discussion

### 2.1. Optimization of the Height of the Modular Solar Tower Reactor

**Figure 1:**
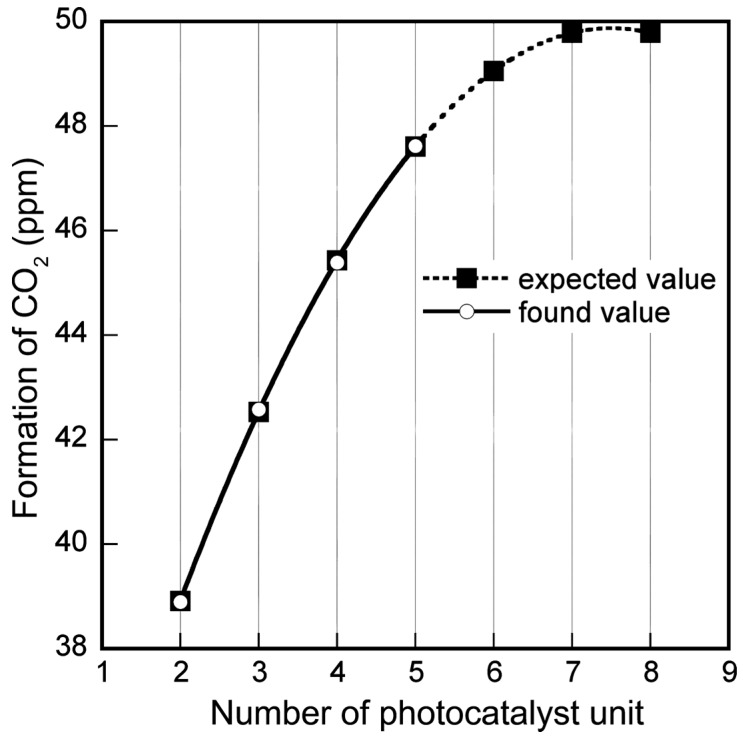
Plot of the amount of CO_2_ formed as a function of the number of photocatalyst units.

To determine the optimum height of the solar tower reactor, the change of the concentration of toluene (and formed CO_2_) under a certain amount of UV radiation was measured as a function of the height of the reactor, which was adjusted by changing the number of photocatalyst modules. Toluene removal and CO_2_ formation exhibited a staircase-like increase with increasing number of units. Photocatalytic degradation of toluene is a well-known reaction, which has been studied through various experiments. Toluene is simply converted to CO_2_ when photocatalysis proceeds stoichiometrically [16]. Figure 1 shows the relationship between the amount of CO_2_ formed and the number of photocatalyst modules. In this experiment, the number of modules was increased from two to five. As shown in Figure 1, the amount of CO_2_ formed as a function of the number of modules was fitted to a second-order polynomial curve. Therefore, a maximum height of 2100 mm (equivalent to seven modules) for the tower was expected to be the best for this system. This estimation shows that UV light, which initiates photocatalytic degradation, reaches the interior of the solar tower reactor to a depth of 2100 mm. Therefore, a height of 2100 mm was chosen for the solar tower reactor.

### 2.2. Elimination of Toluene in the Solar Tower Reactor Installed with a TiO_2_-Coated Ceramic Tube by Solar Irradiation

**Figure 2:**
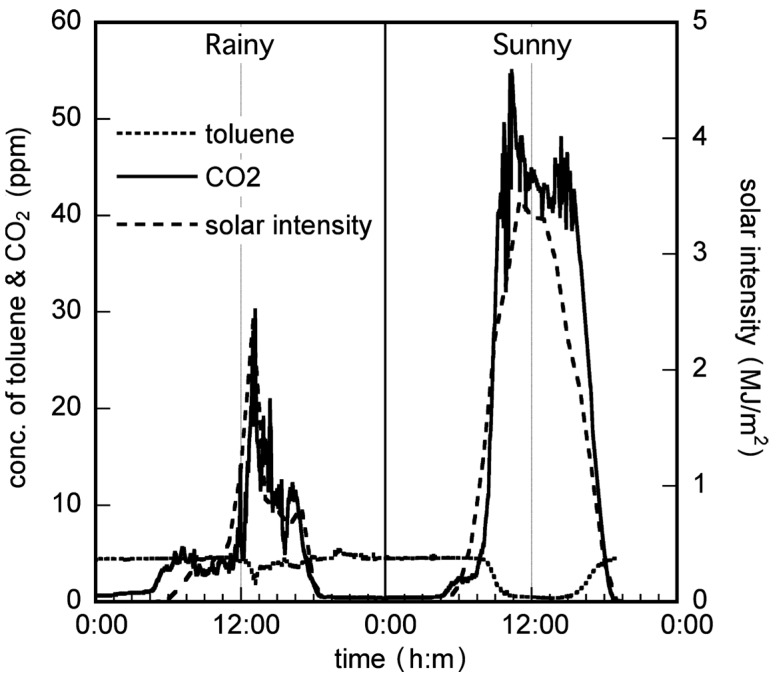
Elimination of toluene in the solar tower reactor installed with a TiO_2_-coated ceramic tube under rainy and sunny conditions.

The data for toluene vapor elimination under sunny and rainy conditions are shown in Figure 2. Under rainy conditions, the initial concentration of toluene (5 ppm) did not change after sunrise. However, UV light from the Sun arrives at the Earth’s surface in spite of rainy conditions. Therefore, CO_2_ formation resulting from the photodegradation of toluene was observed, and its oscillation profile was almost similar to the time profile of solar intensity.

Under sunny conditions, CO_2_ formation began from sunrise, and a decrease in toluene concentration was observed. Within a day, approximately 100% of the toluene was removed, and an almost stoichiometric formation of CO_2_ was observed. Based on these results, CO_2_ formation was considered to depend principally on the intensity of solar radiation, since the oscillation profile of CO_2_ formation was almost similar to the time profile of solar intensity. Under this condition, there was a time lag between toluene degradation and CO_2_ formation, as shown in Figure 2. We assumed that this time lag could be attributed to toluene adsorption onto the TiO_2_ surface before sunrise (dark condition). Toluene introduced to the solar tower reactor was adsorbed onto the TiO_2_ surface at night. This adsorbed toluene was preferentially photodecomposed by solar irradiation instead of the toluene fed to the reactor after sunrise; therefore, the decrease in toluene concentration was not observed at the same time as sunrise. With the increase of solar radiation, the adsorbed toluene was removed by photocatalysis, and the continuously fed toluene started to photodecompose.

**Figure 3:**
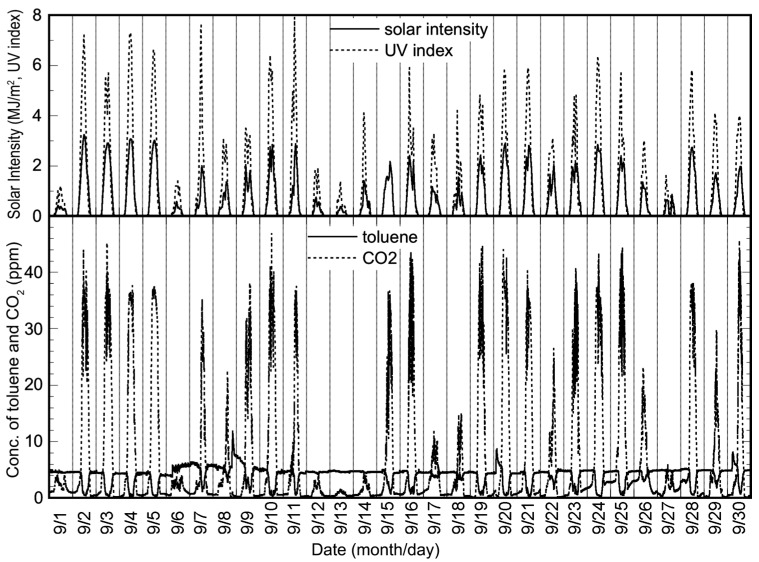
Experimental data for September 2006, obtained using the reactor installed with a TiO_2_-coated ceramic tube and the changes of the temperature.

**Figure 4:**
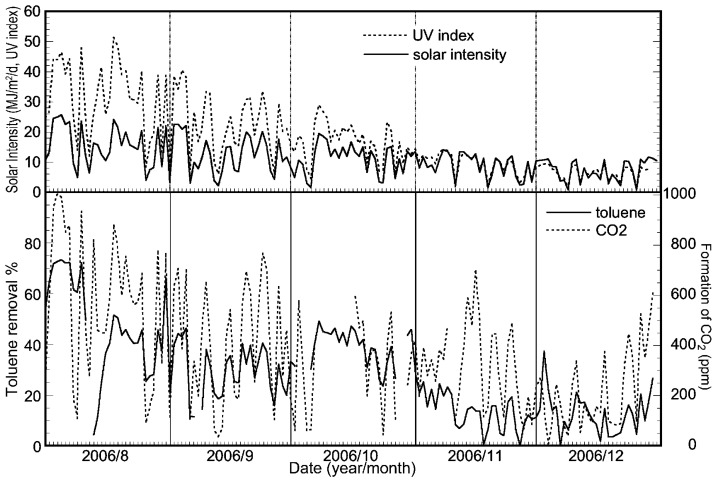
Plots of the integrated values for toluene removal ratio, the concentration of CO_2_ product and the solar intensity per day for five months obtained using the reactor installed with a TiO_2_-coated ceramic tube.

The long-term experiment was carried out from 1 August 2006 to 31 December 2006. Figure 3 shows the experimental data for September as representative of the long-term experiment. It is clear that the toluene removal ratio simply depended on the intensity of solar radiation, as also indicated in Figure 2. Figure 4 shows the plots of the integral values for the toluene removal ratio, the concentration of CO_2_ product and the solar intensity per day for five months. As shown in this figure, the plots of the integrated values for CO_2_ formation and the time profile of solar intensity are almost similar. In addition, the corresponding integrated values are proportional. This shows that there was no decrease in photocatalytic activity during the five months, and a total of 27.7% of the toluene introduced to the solar tower reactor was photocatalytically removed.

### 2.3. Elimination of Toluene in the Solar Tower Reactor Installed with a HQC21-Packed Glass Tube by Solar Irradiation

**Figure 5:**
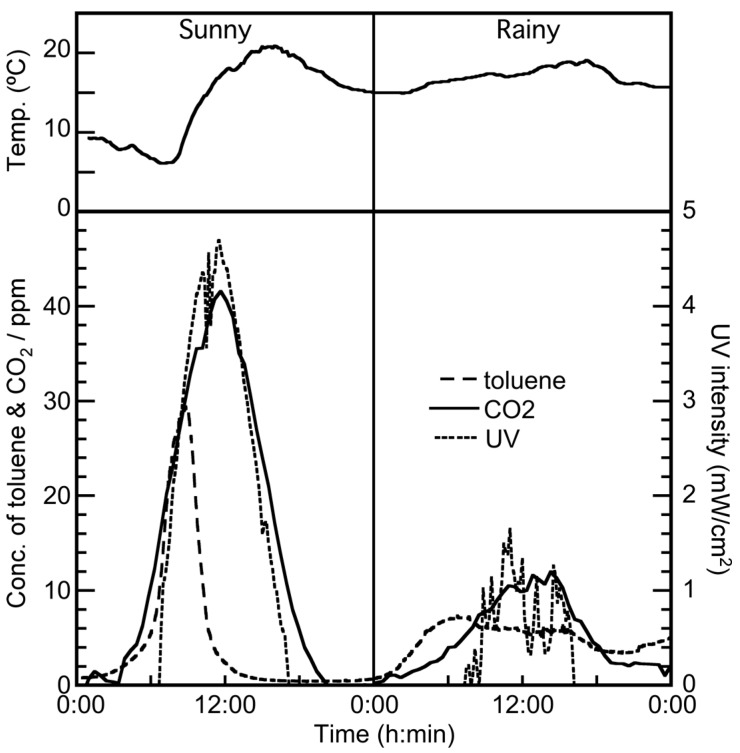
Elimination of toluene by the solar tower reactor installed with a HQC21-packed glass tube under rainy and sunny conditions, and the changes of the temperature.

The data for toluene elimination by the solar tower reactor under sunny and rainy conditions are shown in Figure 5. HQC21 (TiO_2_-coated silica gel produced by Shinto V Cerax) had strong adsorption efficiency, since it was unused, and the silica gel has a wide surface area. Therefore, 5 ppm toluene vapor was easily adsorbed onto HQC21, and the toluene concentration at the outlet of the reactor was constant at 0 ppm until sunrise. The toluene concentration after sunrise reached 30 ppm until 9 AM, after which the concentration began to decrease and was almost 0 ppm at 12 PM. This concentration was maintained until midnight. After 12 AM, the adsorption of toluene began again, and the concentration returned to its initial value at 6 AM, as shown in Figure 5.

The sharp increase in toluene concentration under sunny conditions occurred around sunrise; on the other hand, a correlation between temperature and toluene concentration was not observed, as shown in Figure 5. It was assumed that this sharp increase in toluene concentration indicates desorption of a number of adsorbed toluene molecules from the TiO_2_ surface by solar irradiation. That is, toluene molecules accumulated on the TiO_2_ surface under dark conditions, and desorption of excess toluene began when the adsorbed toluene on the surface of TiO_2_ photodecomposed just after sunrise. Toluene desorption was considerable, and the amount of toluene was higher than the photocatalytic capacity. However, after photocatalysis by solar irradiation, the TiO_2_ surface became free, and consequently, the strong adsorption efficiency of HQC21 was steadily recovered. As shown in Figure 2, strong adsorption of toluene onto the TiO_2_-coated ceramic tube was not observed. It was assumed that this resulted from the difference in the absolute surface area of the photocatalyst between the TiO_2_-coated ceramic tube and the HQC21-packed glass tube. The absolute surface area of the TiO_2_-coated ceramic tube with a total height of 2100 mm was around 11,000 m^2^ (45 m^2^/g × 37 mmD × 300 mm × 7 units). On the other hand, the absolute surface area of the HQC21-packed glass tube with a total height of 1500 mm was around 49,200 m^2^ (200 m^2^/g × 41 g/glass tube × 6 glass tubes) [17].

Under rainy conditions, the adsorption efficiency of toluene onto HQC21 was similar to that under sunny conditions until saturation. However, the concentration of toluene at the outlet of the system did not exceed the inlet concentration, which was constant the whole day. On the other hand, a slight amount of CO_2_ was formed, which indicates that UV irradiation under rainy conditions influenced the photodegradation of adsorbed toluene on the photocatalyst surface.

**Figure 6:**
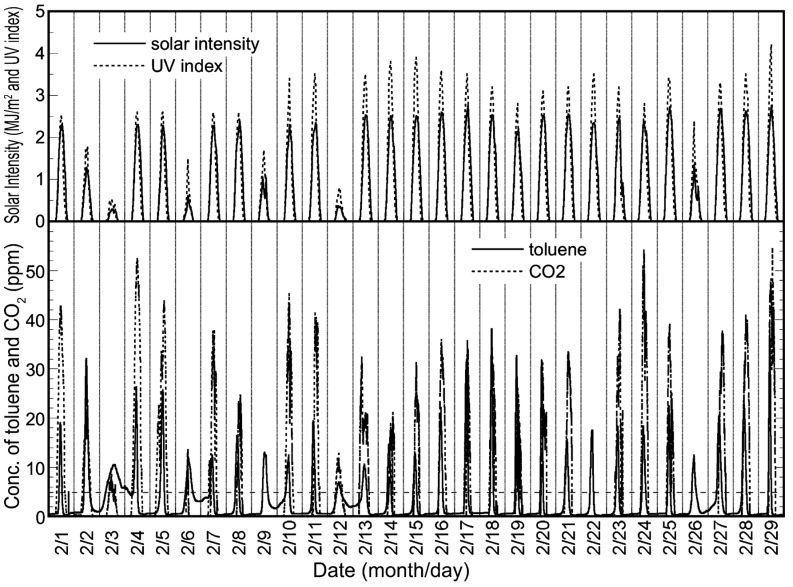
Experimental data for February 2008, obtained using the reactor installed with a HQC21-packed glass tube.

This experiment was conducted for about half a year from 1 January 2008, and the data for February shown in Figure 6 were chosen as representative of this period, because this month had regular, relatively fine weather. As shown in this figure, the outlet concentration of toluene was higher than the inlet one, and it decreased to around 0 ppm in sunny conditions. This trend was observed throughout the half-year experimental period. A higher toluene concentration at the outlet indicates the increase of VOC emissions, and this excess emission to the environment is only a problem in the short term. However, the ratio of the total amount of toluene eliminated to that introduced by the solar tower reactor for the half year reached 36%, which shows that the solar tower reactor is well suited to the long-term reduction of total VOC emission.

**Figure 7:**
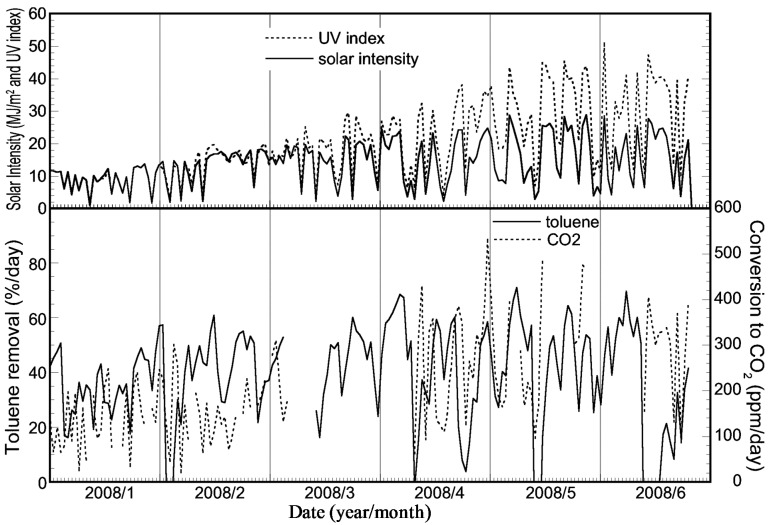
Plots of integrated values for the toluene removal ratio, the concentration of CO_2_ product and the solar intensity per day for five months obtained using the reactor installed with a HQC21-packed glass tube.

Figure 7 shows the changes in integral values for the toluene removal ratio, the amount of CO_2_ and the solar intensity per day for a half year. In this experiment, the CO_2_ data between March to the beginning of April were missing, owing to a malfunction of the CO_2_ monitor. As shown in Figure 7, the profiles of solar intensity, UV index and the amount of CO_2_ are similar. In addition, the emission of CO_2_ also increased proportionally with the progress of the seasons from winter to summer. Therefore, the photocatalytic activity did not decrease during the long trial run of the solar tower reactor.

From the results of these experiments, it is clear that the toluene vapor introduced to the solar tower reactor was eliminated by solar irradiation. On the other hand, the flow rate of toluene vapor in these experiments was still lower than the actual level. Therefore, we will continuously improve the performance of the solar tower reactor by increasing the amount of VOCs eliminated, decreasing the pressure drop and reducing the production cost.

## 3. Experimental Section

### 3.1. Design of the Solar Tower Reactor

**Figure 8:**
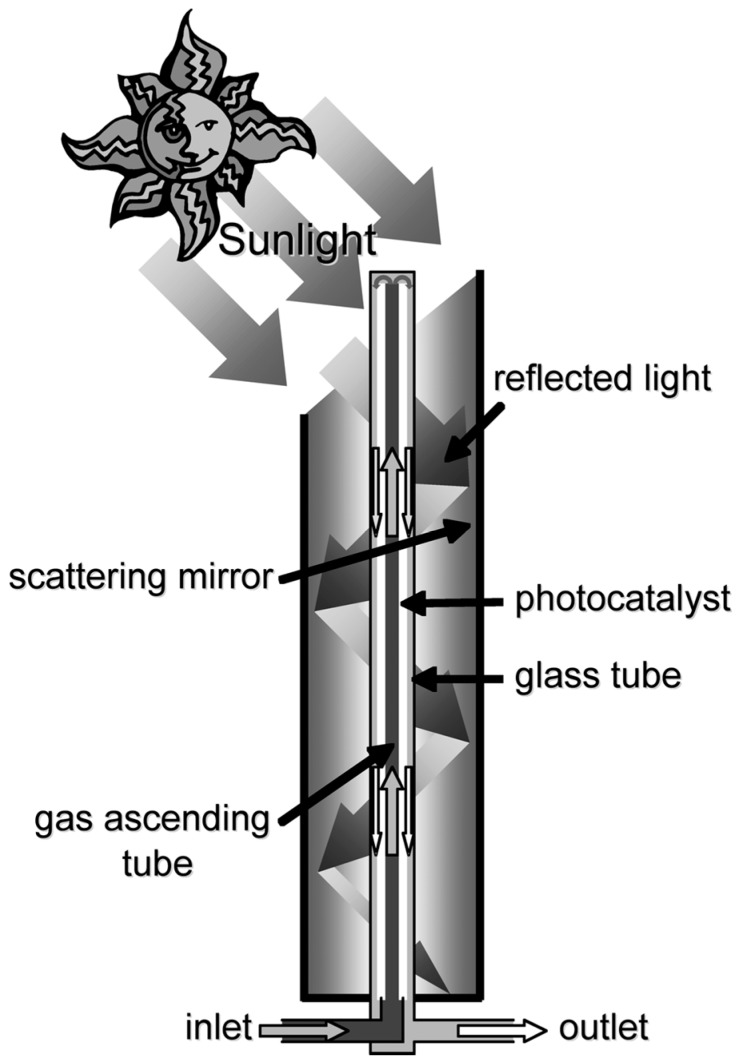
Schematic diagram of the solar tower reactor.

In this section, the structure and operating procedure of the solar tower reactor is described. The tower is shaped like a chimney, and the cylindrical mirror was assembled vertically, as shown in Figure 8. The tubular photocatalyst reactor was placed in its center. The cylindrical mirror was modularized, thus enabling adjustment of the height of the tower to the optimum value by changing the number of modules. Sunlight enters the interior of the cylindrical mirror through an opening at the top. A section of the tubular photocatalyst reactor protrudes above the top of the cylindrical mirror, as shown in Figure 8. The surface of the cylindrical mirror was embossed to scatter incoming solar radiation, and this scattered light reaches the bottom of the tower without concentrating in the center of the cylindrical mirror, the mechanism of which is different from a parabolic trough-type photocatalytic reactor [9]. Simulated VOC gas was supplied once to the top of the photocatalytic reactor, as shown in Figure 8. The top of the reactor was irradiated with direct sunlight, initiating photocatalysis and subsequent reduction of the initial concentration of the VOCs. The intensity of the light incident through the opening at the top decreases as repeated scattering and reflections go downward. However, the VOC concentrations are also continuously decreased by photocatalysis during the downward gas flow; therefore, the weakened light intensity is strong enough for the photocatalysis of residual VOCs. This mechanism allows continuous photocatalysis of the VOCs by the solar tower reactor. The top of the tubular mirror was diagonally cut for effective collection of solar radiation for the whole day. The angle of the cylindrical mirror of the sunlight entrance was determined from the following equation,

Angle of the cylindrical mirror of the sunlight entrance (°) = 45° + 0.5 × latitude
(1)

There is a small deviance between the Earth’s equatorial plane and the ecliptic plane; thus, this equation is not precisely applicable. However, this simple equation is sufficient for calculating the maximum sunlight intensity in a whole year.

### 3.2. Photocatalysts

Two types of photocatalytic materials were used in the experiment. One was a TiO_2_-coated ceramic tube with an external diameter of 37 mm and a length of 300 mm, and the other was a TiO_2_-coated silica gel (HQC-21; purchased from Shinto V Cerax, Aichi, Japan) packed in a Pyrex^®^ glass tube with an internal diameter of 10 mm and a length of 1500 mm. A single flow pass was employed for both types, as shown in Figure 8.

**Figure 9:**
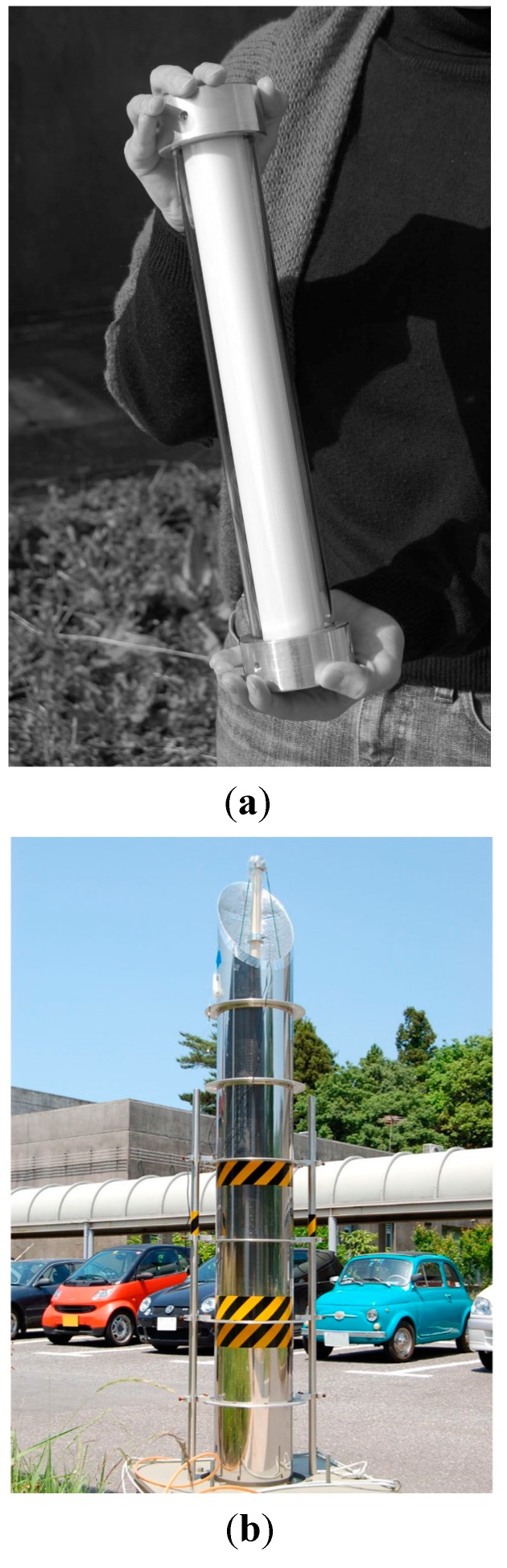
(**a**) Module of the TiO_2_-coated ceramic tube inside a Pyrex^®^ glass tube; (**b**) external view of the solar tower reactor assembled from these modules.

**Figure 10:**
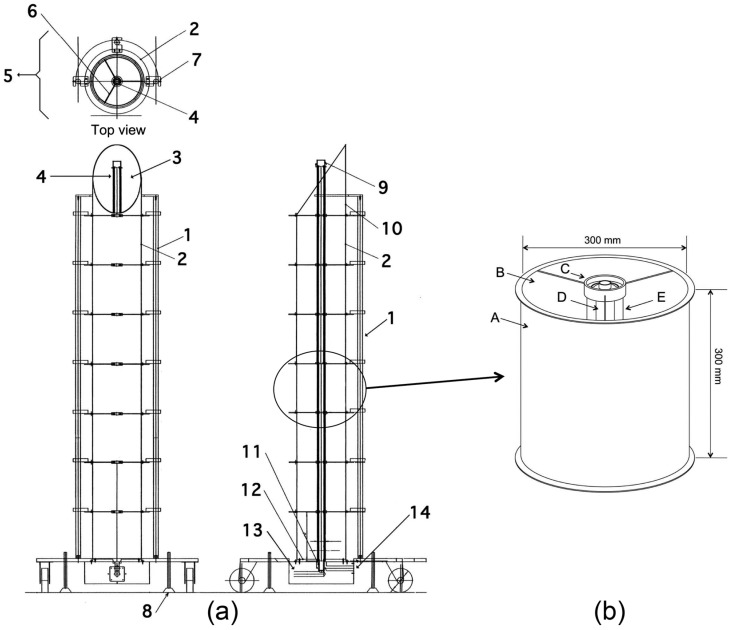
(**a**) Diagram of the solar tower reactor. 1: support rod; 2: cylindrical mirror; 3: embossed scattering mirror; 4: photocatalyst; 5: top view; 6: shaft carrier; 7: connector; 8: anchor; 9: gas return cap; 10: entry point of solar light; 11: inlet/outlet separator; 12: embossed scattering mirror; 13: air inlet; 14: air outlet. (**b**) Module of the solar tower reactor. A: cylindrical mirror; B: embossed scattering mirror; C: glass cover connector; D: TiO_2_-coated ceramic tube; E: Pyrex glass cover.

The preparation of the TiO_2_-coated ceramic tube was carried out using the dip coating method [18]. A γ-alumina tube with an external diameter of 37 mm, an internal diameter of 30 mm and a length of 300 mm was used as the substrate for TiO_2_ coating. The TiO_2_ precursor was dip-coated onto this ceramic tube, and the TiO_2_ thin film photocatalyst was obtained by calcination at 480 °C for 1 h. The coating procedure was repeated 18 times to obtain the final photocatalytic material. The film thickness of the TiO_2_ layer on the ceramic tube was impossible to measure, except through destructive methods; therefore, the thickness was estimated from data obtained in our previous study [18]. It was shown that a single coating step yields a 200 nm-thick film. Therefore, the estimated film thickness of the TiO_2_ film on the ceramic tube was approximately 3.6 µm for the 18 cycles of dip coating. Seven TiO_2_-coated ceramic tubes were prepared, and each was placed inside a Pyrex^®^ glass tube with an internal diameter of 49 mm, as shown in Figure 9a. This photocatalytic module was fitted with metal molding adapters to enable a connection with other modules. The photocatalytic material is placed in the center of the cylindrical mirror of the solar tower reactor. The external view is shown in Figure 9b. A diagram of the solar tower reactor is shown in Figure 10a. The cylindrical mirror that constitutes the reactor was modularized, as shown in Figure 10b, and the height of the tower can be adjusted to the optimum value by changing the number of modules.

**Figure 11:**
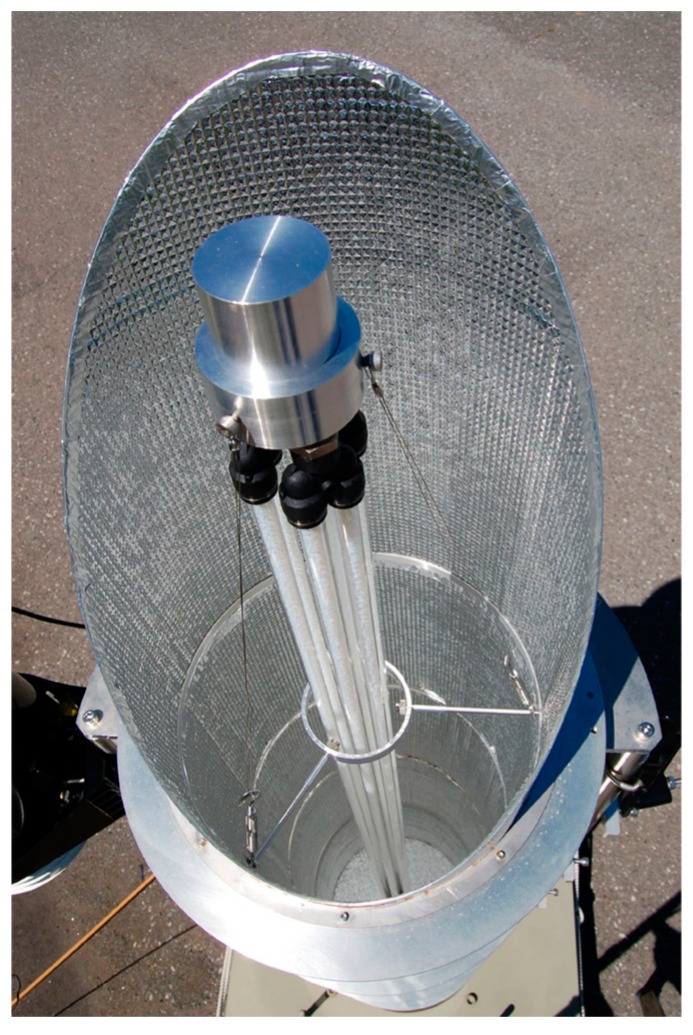
Top view of the HQC21-packed glass tube reactor.

The glass tube packed with TiO_2_-coated silica gel was prepared as follows. HQC21 (average diameter = 4 mm, specific surface area = 200 m^2^/g, average TiO_2_ deposition onto silica gel = around 20 wt %) [19] was used as TiO_2_-coated silica gel. The dimensions of the Pyrex^®^ glass tube were 12 mm external diameter, 10 mm internal diameter and 1500 mm length. Six glass tubes packed with HQC21 were prepared, each containing 41 g of HQC21. The HQC21-packed glass tubes were arranged at the center of the solar tower reactor, as shown in Figure 11.

### 3.3. Construction of the Gas Flow System for Evaluating the Performance of the Solar Tower Reactor

**Figure 12:**
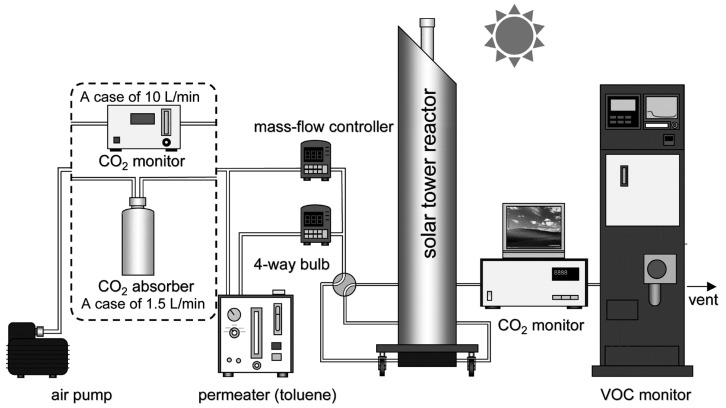
Scheme of the gas flow system for the evaluation of the performance of the solar tower reactor.

Figure 12 shows the gas flow system constructed to evaluate the performance of the solar tower reactor. The air pump pulled in ambient air, which was then separated into two directions. One route led to the permeater for the generation of toluene vapor, while the other one led directly to the mass flow controller, where the normal air acted as the carrier gas. Toluene vapor and normal air were mixed through the mass flow controller, and 5 ppm toluene vapor was obtained. A different flow rate for the toluene vapor was used for each type of photocatalyst reactor. A flow rate of 1.5 L/min was used for the TiO_2_-coated ceramic tube reactor; on the other hand, a flow rate of 10 L/min was used for the HQC21-packed glass tube reactor. To measure the toluene vapor concentration, the VM500 online VOC monitor (Yokogawa Inc., Tokyo, Japan) was used. It is well known that the humidity in air is a principal factor for photocatalysis, *i.e.*, photocatalysis of toluene requires humidity [20,21,22,23]. However, the humidity was not controlled in this experiment. One of the reasons is that we needed to simulate the actual operation condition. Obee *et al.* reported that the photocatalytic oxidation rate of toluene under low humidity was extremely low, and the high oxidation rate of toluene was indicated at around 20% relative humidity. The rate was gradually decreased with the increase in relative humidity until 90% [20]. The concentration of CO_2_ produced from the photodecomposition of toluene was measured by the Thermo 41C CO_2_ monitor (Thermo Fisher Scientific Inc., Waltham, MA, USA). When the flow rate used was 1.5 L/min, CO_2_ in the ambient air was removed by 500 g soda lime (Wako Chemical, Osaka, Japan). On the other hand, an additional CO_2_ monitor (LX-720, Iijima-Denshi Co., Aichi, Japan) was connected to the outlet of the solar tower reactor instead of the CO_2_ adsorbent when the flow rate was 10 L/min. In this case, the concentration of CO_2_ was calculated from the difference between the outlet and inlet concentrations at the solar tower reactor. Data were collected using a personal computer (PC) connected to both CO_2_ monitors. During the experiment, some CO_2_ data could not be collected, owing to malfunctions of either the CO_2_ monitor or the PC.

The pressure drop within the Pyrex^®^ tube packed with TiO_2_-coated silica gel was quite high compared with that of the TiO_2_-coated ceramic tube; however, the actual values were not measured in the experiment. On the other hand, the residence time of toluene vapor in the reactor was calculated. The photocatalytic module made from the TiO_2_-coated ceramic tube was annular, with an internal volume of 243 mL (Figure 9b). As the flow rate in this module was 1.5 L/min, the residence time of toluene vapor was around 9.7 s. The total residence time in the solar tower reactor was obtained by multiplying this value with the number of modules. For example, for 7 modules (*i.e.*, height of 2100 mm), the total residence time of the toluene vapor was around 68 s. In the case of the HQC21-packed glass tube, the free volume was around 64 mL, assuming the closest packing of particles with a 4 mm diameter into the glass tube having an internal diameter of 10 mm and a length of 1500 mm. There were 6 HQC21-packed glass tubes; therefore, the total free volume in the solar tower reactor was around 385 mL. The total residence time of the toluene vapor was around 2.3 s at a flow rate of 10 L/min.

### 3.4. Optimization of the Height of the Solar Tower Reactor

**Figure 13:**
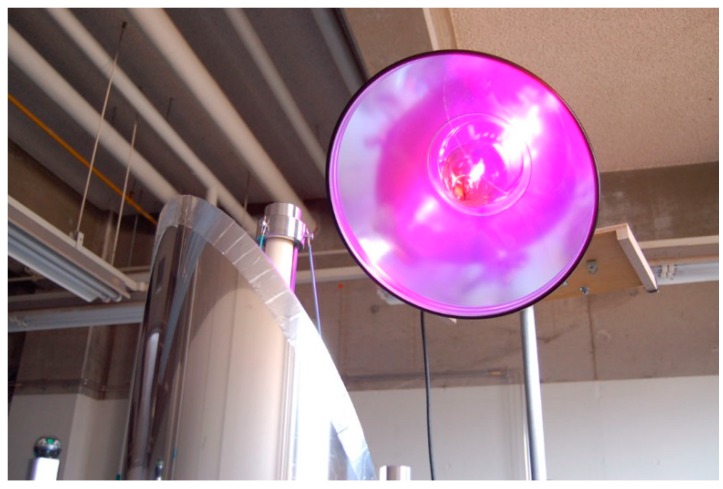
Artificial UV-A lamp.

The optimum height of the solar tower reactor was determined from the variation of the toluene removal ratio with the number of TiO_2_-coated ceramic tube modules. For this experiment, an artificial 400-W UV light (H400L-BL, Iwasaki Electric Co., Tokyo, Japan) was used (Figure 13). This procedure was not applied for the HQC21-packed glass tube reactor, because the corresponding experiment was only performed using the standard length of the Pyrex^®^ glass (1500 mm). The intensity of the UV light at the top of the solar tower reactor was 3.5 mW/cm^2^ (λ = 365 nm).

### 3.5. Evaluation of the Performance of the Solar Tower Reactor 

Evaluation of the solar tower reactor using the TiO_2_-coated ceramic tube was performed for 5 months from 1 August 2006 to 31 December 2006. Evaluation of the solar tower reactor using the HQC21-packed glass tube was performed for 6 months from 1 January 2008 to 24 June 2008. For the experiments done in 2006 and 2008, the intensity of sunlight was expressed in terms of the insolation and UV index. Insolation data from the Aerological Observatory/Japan Meteorological Agency (JMA) [24] were used, because JMA is adjacent to the Advanced Industrial Science and Technology (AIST), Tsukuba. For the 2008 experiment, the temperature was measured by Vantage Pro 2 (Davis Instruments Corp., Hayward, CA, USA).

## 4. Conclusions

We constructed a solar tower reactor for the elimination of low concentrations of VOCs, such as toluene. Two types of photocatalytic units were used: (1) a TiO_2_-coated ceramic tube optimized for a low pressure drop and low flow rate (1.5 L/min); and (2) a glass tube packed with TiO_2_-coated silica gel (HQC21) optimized for a high pressure drop and high flow rate (10 L/min). Both reactors effectively eliminated toluene vapor (5 ppm); however, the toluene removal profile was different between the TiO_2_-coated ceramic tube and HQC21-packed glass tube reactor. The time profile of toluene removal by the TiO_2_-coated ceramic tube reactor was almost similar to that of solar intensity. On the other hand, the toluene concentration at the outlet was temporarily higher than the inlet concentration at sunrise in the case of the HQC21-packed glass tube reactor. This difference between the two reactors was attributed to the difference in the actual surface area of the photocatalyst. Toluene vapor introduced to the reactor adsorbed and accumulated onto the TiO_2_ surface at night, and this adsorbed toluene desorbed from the TiO_2_ surface once it was by irradiated by sunlight. The difference between the specific surface areas of TiO_2_ in the two reactors was not considerable; however, the total amount of photocatalyst in the HQC21-packed glass tube reactor was higher than that in the TiO_2_-coated ceramic tube reactor. In either case, the long-term experiment conducted for a half year demonstrated that toluene vapor introduced into the solar tower reactor was eliminated by solar irradiation by an amount proportional to the integral solar power intensity.
